# A Text-Book of Pathology

**Published:** 1901-10

**Authors:** 


A TEXT-BOOK OF PATHOLOGY. By Alfred Stengel, M. D., Professor of Clinical Medicine in the University of Pennsylvania, Etc. With 372 illustrations. Third edition, revised. Pages, 873. Price, $5.00. Philadelphia and London: W. B. Saunders & Co. 1900.

The author intended to supply a moderate sized book on clinical pathology—a book that could be handled easily and sufficiently elaborate to embrace all the facts without the cumbersome details so often included in text-books on this subject. That this has been accomplished by the author can be verified by an examination of the book. It is a thoroughly up to date work.

Part I is devoted to general pathology, including the etiology of disease, disorders of nutrition and metabolism, disturbances of the circulation of the blood, the retrogressive processes, inflammation and regeneration, progressive tissue changes, etc. There is a chapter devoted to bacteria and the diseases due to bacteria, and also one devoted to animal parasites and diseases caused by them.

Part II is devoted to special pathology, including diseases of the blood, lymphatic tissues, the circulatory and respiratory systems, the gastro-intestinal tract, the ductless glands, the urinary organs, the reproductive organs, the bones, joints, muscles, the brain and membranes, the spinal cord and nervous system. T. J. B.

